# Missed diagnoses and misdiagnoses of adults with autism spectrum disorder

**DOI:** 10.1007/s00406-020-01189-w

**Published:** 2020-09-06

**Authors:** Laura Fusar-Poli, Natascia Brondino, Pierluigi Politi, Eugenio Aguglia

**Affiliations:** 1 Department of Clinical and Experimental Medicine, Psychiatry Unit, University of Catania, via Santa Sofia 78, 95123 Catania, Italy; 2 Department of Brain and Behavioral Sciences, University of Pavia, via Bassi 21, 27100 Pavia, Italy

**Keywords:** Autism spectrum disorder, Adults, Psychiatry, Sex, Female phenotype, Diagnosis

## Abstract

Autism spectrum disorder (ASD) is a group of life-long neurodevelopmental disorders affecting 1.5% of the general population. The present study aimed to evaluate the psychiatric history of a group of adults who received the first diagnosis of ASD in two Italian university centers. Diagnoses of ASD were confirmed by a team of psychiatrists with wide expertise in the field, after the administration of standardized tools (i.e., ADOS-2, ADI-R). The sample comprised 161 participants, of which 114 (79.5%) were males. The median age of diagnosis was 23 years (range 18–55), with a median IQ of 100 (range 30–145). The first evaluation by a mental health professional was performed at a median age of 13 years, with a gap of 11 years between the first evaluation and the diagnosis of ASD. 33.5% of participants had never received a psychiatric diagnosis, while the rest of the sample had received one or more diagnoses different from ASD. The most common past diagnoses were intellectual disability, psychoses, personality disorders, and depression. Sex differences were detected in the age of diagnosis and ADOS-2 scores. Our results provide important information for both child and adult psychiatrists. Given the prevalence of autism and the high rates of co-occurrent psychiatric conditions, it is important for clinicians to consider ASD in the differential diagnostic process.

## The “uniformologist”

A. had been followed by the community psychiatric services for several years with a diagnosis of personality disorder not-otherwise-specified in comorbidity with bipolar disorder. He was a funny and lively 55-year-old man, with scarcely modulated eye contact. The way of speaking was polished and verbose. It was possible to get in contact with A., even if it was very difficult to stop him while talking about his special interests. In fact, A. showed an overpowering passion for history, knowing almost every detail of historical events. In particular, he was fascinated by uniforms (“I am an expert in uniformology”, he said). Such pervasive passions caused difficulties to A. in the management of daily life, since he spent much time of the day pursuing his interests.

He was living alone in a flat next to his old father, but was not able to attend to living activities autonomously. The municipality daily provided the lunch to A., while the dinner was usually prepared by his sister. Additionally, a housekeeper helped him cleaning the house. A. did not have a stable job, only a seasonal employment as cultural operator. He had obtained a master’s degree in history and, in the past, he had worked as a teacher in high schools, never being able to maintain the job for long periods and encountering several difficulties in managing the classrooms. A. had also problems in the interaction with other people, not always interested in his preferred topics, and his relationships were limited to his seasonal job.

A.’s old father and sister reported complications during birth, but a regular development, without noticeable delays. They also referred that, during childhood, he was diagnosed with epilepsy for which he was still taking medication. Additionally, they reported that during several years, the patient had personally written dozens of books regarding historical themes of his interest, collecting information from different sources. However, such masterpieces were hidden, and no one had access to them.”

## Introduction

Recent data have estimated that around 1.5% of the population in developed countries is affected by autism spectrum disorder (ASD), a group of lifelong neurodevelopmental conditions usually diagnosed during early childhood [1]. Prevalence of ASD has been constantly growing since its first description by Leo Kanner [2], mainly because of the growing awareness among the general population and the scientific community [3]. Moreover, after the release of the 5th Edition of the Diagnostic and Statistical Manual of Mental Disorders (DSM-5), changes in diagnostic criteria have introduced the concept of “spectrum” and acknowledged the possibility of diagnosing ASD also in individuals whose deficits do not "become fully manifest until social communication demands exceed limited capacities" [4], for instance during adolescence or adulthood. For this reason, many professionals worldwide are now trying to identify the “lost generation” of adults with ASD [5].

Diagnosing ASD in adulthood may be difficult for clinicians, for several reasons. The first challenge is related to the difficulties encountered in gaining information about the developmental history. Parents or caregivers, in fact, could be unavailable, or the trustworthiness of provided information might be poor due to the time elapsed between infancy and the clinical assessment [6]. Moreover, adults with ASD, particularly those with higher intelligence quotient (IQ), may develop coping and camouflaging strategies. In some cases, these strategies are present since childhood, thus making it difficult for parents or caregivers to capture underlying difficulties. Additionally, they may represent an obstacle for clinicians during a formal assessment, as core symptoms might be masked, hampering a correct identification of ASD. For instance, ASD adults may have learned to “look into the eyes” and only subtle differences in eye contact modulation and integration with verbal behavior may be evident [6]. As acknowledged by the DSM-5 itself, autistic individuals with high cognitive abilities and effective coping strategies may not present substantial impairments until adolescence or adulthood, therefore never seeking a clinical diagnosis or support [4, 7].

The risk of going undiagnosed is even more elevated for women on the autism spectrum [5, 8]. This is shown by the observation that in non-referred samples, there are two-to-three males for each female with ASD [9, 10], whereas in clinical samples the male-to-female ratio is usually four-to-one or higher [1]. Also, females are frequently diagnosed later than their male peers [11]. This seems related to several factors, such as the standardization of diagnostic tools on male samples, but also the higher degree of camouflaging and coping strategies. Moreover, women usually present more internalizing than externalizing symptoms, which might be easily confused with anxiety or depression and may not be noticed by caregivers or clinicians [12]. The DSM-5 itself acknowledges that “girls without accompanying intellectual disability or language delays may go unrecognized, perhaps because of subtler manifestation of social and communication difficulties” [4].

Adults in the autism spectrum may remain unrecognized for different reasons. First, they may have never referred to child or adult psychiatric services (i.e., missed diagnoses). Second, they have been incorrectly diagnosed with other psychiatric disorders over the course of life; in fact, symptoms of ASD overlap with those of other psychopathological conditions, such as personality disorders, psychoses, anxiety disorders, obsessive–compulsive disorders (OCD) and intellectual disability (ID) [13], thus making ASD less identifiable by clinicians who are not familiar with the condition (i.e., misdiagnoses). Third, the psychiatric disorder in question may be present in comorbidity with the autistic condition, thus partially covering ASD core symptoms (i.e., psychiatric comorbidity). Indeed, a recent meta-analysis evaluating the prevalence of co-occurring mental health diagnoses in the autism population has found a pooled prevalence of 20% for anxiety disorders, 11% for depressive disorders, 9% for OCD, 5% for bipolar disorders, and 4% for schizophrenia spectrum disorders [14], suggesting that psychiatric issues are more common in people with ASD than the neurotypical population.

Original studies have reported that adults who were seeking a first ASD diagnosis had been frequently diagnosed with other psychiatric disorders, such as anxiety, attention deficit–hyperactivity disorder (ADHD), mood disorders, and personality disorders [7, 15]. In a recent systematic review, Tromans and colleagues [16] focused on unrecognized autistic adults among psychiatric inpatients, estimating a prevalence between 2.4 and 9.9%, as reported by four studies [17]. Analogously, Nylander and Gillberg [18] found that 3.2% of patients attending a treatment center for severe psychiatric disabilities had ASD. Chang et al. found a prevalence of 0.6% of unrecognized or misdiagnosed adults with ASD in a psychiatric outpatient clinic in Taiwan [19]. Similar findings were reported by Davidson et al., who reported that 2.6% of patients referred to an early intervention service for psychosis had Asperger syndrome [20]. Finally, Geurts and Jansen [15] showed that most adults with ASD were known within the mental health system before they received a specific assessment for ASD. The time elapsed between the first contact with services and the ASD assessment ranged from 0 to 56 years (median 12 years) [15].

In the present retrospective study, we will summarize the characteristics of 161 adults who were referred to two Italian university centers after the release of DSM-5 and obtained the first formal diagnosis of ASD. Then, we will revise the demographic characteristics and the scores obtained at standardized tests. A specific focus will be given to the psychiatric history of participants, with a detailed examination of the number and type of previous diagnoses, as well as the time elapsed from the first referral to mental health services to the ASD diagnosis. Finally, we will discuss the sex differences detected through the analysis of our sample.

## Material and methods

### Procedures

We retrospectively reviewed the clinical charts of all adults who received a first formal diagnosis of ASD after the publication of the DSM-5 [4] and up to December 2019. Participants were evaluated in two Italian university services specialized in the assessment and treatment of adolescents and adults with ASD (Laboratorio Autismo, University of Pavia; Outpatient service for ASD, Policlinico University Hospital, Catania). To be included in the present study, participants had to fulfill the following criteria: being 18 years or older at the moment of first evaluation in our centers; having never received a diagnosis of ASD in the past; signing a written informed consent.

People referred to our centers (or their guardians) always sign a written informed consent during the first visit, allowing the use of their data for research purposes. Thus, written informed consent was obtained for inclusion in the present study. The informed consent was signed by 121 participants in person, while guardians signed on behalf of the remaining 40 participants. The protocol was approved by our internal review board. The study was performed in accordance with the Declaration of Helsinki.

### Clinical assessment

All participants were assessed with identical procedures by the clinical staff of each of the two facilities, composed of at least one senior psychiatrist and one trainee in psychiatry, all with wide expertise in the assessment and treatment of people with ASD. Information regarding developmental and personal history, as well as previous clinical evaluations, was collected. Particular care was given to the age of first evaluation by a mental health professional (i.e., child or adult psychiatrist, psychologist) and to previous psychiatric diagnoses, which were annotated in a specific database using the 10th Edition of the International Classification of Diseases (ICD-10) codes [21]. According to routine psychiatric practice, all participants underwent an exhaustive clinical evaluation, specifically focused on ASD, but which also considered possible differential diagnoses and co-occurring psychiatric conditions. Standardized tools for the diagnosis of ASD and for the evaluation of intelligence quotient (IQ) were administered to all participants (see next paragraph), while the assessment of comorbidities and severity was based on the clinical history, mental state examination, and informants’ reports.

Final ASD and co-occurrent psychiatric diagnoses were performed by consensus among all members of the clinical staff, according to the DSM-5 criteria. Severity levels were specified for each of the two main diagnostic criteria (criterion A: deficits in socio-communication; criterion B: presence of restricted interests or stereotypies). According to the DSM-5, level 1 requires support, level 2 requires substantial support, while level 3 autism requires very substantial support [4].

### Standardized instruments

Standardized instruments or interviews specifically developed for the assessment of ASD in adulthood, such as the Autism Diagnostic Observation Schedule-2 (ADOS-2) [22] and the Autism Diagnostic Interview-Revised (ADI-R) [23] were administered to participants or their caregivers.

The ADOS-2 is a semi-structured observation of individuals who may belong to the autism spectrum [22]. It is composed of different domains: Communication, Reciprocal Social Interaction, Communication + Social Interaction, Imagination/Creativity, and Stereotyped Behaviors and Restricted Interests (RRB). The ADOS-2 consists of five modules addressed to children and adults according to their developmental and language levels. For the purpose of the present study, only individuals with good verbal fluency were administered ADOS-2 Module-4 (*n* = 138). As proposed by Lord et al. in the original diagnostic algorithm [22], we considered the ADOS-2 suggestive of a diagnosis of ASD if the subject met the cutoff values for the autism spectrum in the Communication domain (score of 2 or above), Social Interaction domain (4 or above), as well as in the Communication + Social Interaction domain (7 or above). Imagination/Creativity and RRB domains were not considered in the final scoring.

The ADI-R is a semi-structured interview that covers all three major areas of impairment in autism [quality of reciprocal social interaction; communication; repetitive, restricted, and stereotyped patterns of behavior (RRB)] [23]. A prominent part of the interview focuses on the period between the ages of 4 and 5 years, when differences among individuals with different levels of functioning can be better observed and compared. Since the interview should be administered to parents or childhood caregivers, it was scored only for 130 participants. The ADI-R was considered positive for a diagnosis of ASD if the scores in the three domains exceeded the cutoff values. As proposed by Lord [23], the total cutoff score for the Communication domain was 8 for verbal subjects and 7 for non-verbal subjects. For all individuals, the cutoff for the Social Interaction domain was 10, and the cutoff for the Restricted and Repetitive Behaviors domain was 3.

Moreover, all individuals underwent an evaluation of the intelligence quotient (IQ), by means of available tools (i.e., WAIS-R, Leiter-3 or Raven’s Matrices) to evaluate the presence of intellectual disability (ID). Participants were considered as having ID if they scored less than 70 at standardized tools.

Other instruments, such as the Hamilton Depression Rating Scale (HAM-D), Hamilton Anxiety Rating Scale (HAM-A), Structured Clinical Interview for DSM-IV Axis II Disorders (SCID-II), and Minnesota Multiphasic Personality Inventory-2 (MMPI-2), were administered only in case of diagnostic uncertainty.

The severity of ASD was assessed on the basis of clinical evaluation and informants’ accounts. Specific tools for the evaluation of adaptive abilities [e.g., Vineland Adaptive Behaviors Scales-II (VABS), or Adaptive Behavior Assessment System (ABAS-II)], due to time restrictions and availability of personnel and participants, were administered only in case a disability certificate was needed. Results of the scales administered for differential diagnosis, comorbidities, and adaptive abilities were described in clinical reports, but not stored in a database, and thus cannot be reported in the present paper.

### Statistical analysis

Data were tested for normal distribution and homogeneity of variance using Kolmogorov–Smirnov and Levene’s tests before statistical procedures were applied. As the distribution was not normal for all variables, descriptive statistics were presented as median and ranges for continuous variables or counts and percentages for categorical variables. The Mann–Whitney *U* test was used to calculate differences between males and females for continuous variables, while the Chi-squared and Fisher’s exact tests were used for categorical variables. Effect sizes for Mann–Whitney *U* test were expressed as r, which was calculated using the formula *r* = *z*/√ *n* [24]. For Chi-squared and Fisher’s exact test, the effect size was reported as Cramér's *V* (*φ*_c_). The effect sizes were interpreted according to Cohen’s guidelines: 0.1 was considered a small effect, 0.3 a medium effect, and 0.5 a large effect [24]. To avoid type I errors (false positives) and decrease the false discovery rate (FDR), we have adjusted *p* values using the Benjamini–Hochberg procedure [25]. Differences were regarded as significant for *p* values < 0.05. All statistical analyses were performed using IBM SPSS Statistics v. 23.

## Results

### General characteristics of the sample

We reviewed the clinical charts of 161 people who requested a formal assessment for ASD. Our sample was composed mainly of males (79.5%) and had a median age of 23 years (range 18–55) at the time of our evaluation and diagnosis. According to Mann–Whitney test, women were diagnosed significantly later than men (*U* = 1700, *r* = 0.29, *p* < 0.001, adj *p* = 0.003). Median IQ was in the normal range (100). 20.5% of participants had comorbid ID. Median IQ was slightly higher in females than males (110 in females; 97 in males), but the difference was not statistically significant after correcting for FDR (*U* = 2128, *r* = 0.16, *p* = 0.04, adj *p* = 0.10). Severity levels were mainly 1 or 2 for both criteria. A minor proportion of participants required a substantial level of support, specifically 14.3% for criterion A and 8.1% for criterion B. No significant differences were found in severity levels according to sex, neither for criterion A (*χ*^2^ = 2.05, *φ*_c_ = 0.11, *p* = 0.36, adj *p* = 0.41) nor criterion B (χ^2^ = 3.61, φ_c_ = 0.15, p = 0.16, adj p = 0.24). Participants were mainly referred by families (42.2% of the sample), by other clinicians (35.4%) or were self-referred (22.4%), with no significant differences between males and females (*χ*^2^ = 3.08, *φ*_c_ = 0.14, *p* = 0.21, adj *p* = 0.28).

As for psychiatric comorbidities, the most frequently present ones at the time of ASD formal evaluation and diagnosis were depression (9.9%) and anxiety (6.2%), following which we detected psychosis (4.3%), OCD (3.1%), and learning disabilities (2.5%). Other co-occurrent psychiatric conditions were found only in a small proportion of participants. The characteristics of the overall sample are presented in Table 1.

**Table 1:** General characteristics of adults with autism spectrum disorder (ASD)

Variable	Total (*n* = 161)	Males (*n* = 114)	Females (*n =* 47)	*U*/*χ*^2^	*r*/*φ*_c_	*p* value	Adj *p* value
Age, median (range)	23 (18–55)	22 (18–55)	26 (19–51)	1700	0.29	** < 0.001**	**0.003**
IQ total, median (range)	100 (30–145)	97 (30–145)	110 (50–145)	2128	0.16	**0.04**	0.10
Intellectual disability, *n* (%)	33 (20.5)	25 (21.9)	8 (17)	0.49	0.05	0.48	0.53
Severity A, *n* (%)				2.05	0.11	0.36	0.41
Level 1	82 (50.9)	54 (47.4)	28 (59.6)				
Level 2	56 (34.8)	43 (37.7)	13 (27.7)				
Level 3	23 (14.3)	17 (14.9)	6 (12.8)				
Severity B, *n* (%)				3.61	0.15	0.16	0.24
Level 1	85 (52.8)	55 (48.2)	30 (63.8)				
Level 2	63 (39.1)	48 (42.1)	15 (31.9)				
Level 3	13 (8.1)	11 (9.6)	2 (4.3)				
Referral, *n* (%)				3.08	0.14	0.21	0.28
Self-referred	36 (22.4)	25 (21.9)	11 (23.4)				
Family	68 (42.2)	44 (38.6)	24 (51.1)				
Other clinicians	57 (35.4)	45 (39.5)	12 (25.5)				
Psychiatric comorbidities, *n* (%)	48 (29.8)	31 (27.2)	17 (36.2)	1.28	0.09	0.26	0.33
Depression	16 (9.9)	8 (7)	8 (17)				
Anxiety	10 (6.2)	7 (6.1)	3 (6.4)				
Psychoses	7 (4.3)	5 (4.4)	2 (4.3)				
Obsessive–compulsive disorder	5 (3.1)	4 (3.5)	1 (2.1)				
Learning disabilities	4 (2.5)	4 (3.5)	0 (0)				
Others	7 (4.3)	3 (2.6)	4 (8.5)				

*Adj p-value *Benjamini–Hochberg adjusted *p* value, *IQ* intelligence quotient. Significant *p* values and adjusted *p* values are marked in bold

The ADOS-2 was administered to 138 out of 161 participants. After correcting for FDR, a significant difference was found in the Social Interaction (*U* = 1272.5, *r* = 0.30, *p* = 0.001, adj *p* = 0.008), Communication + Social Interaction (*U* = 1324.5, *r* = 0.27, *p* = 0.001, adj *p* = 0.006), and RRB (*U* = 1400.5, *r* = 0.25, *p* = 0.003, adj *p* = 0.01) domains, with scores higher in males than females. The ADOS-2 was above the cutoff for the original algorithm in 89.8% of the sample, with significant sex differences (*χ*^2^ = 17.05, *φ*_c_ = 0.35, *p* < 0.001, adj *p* < 0.001). In fact, the males included in our sample scored above the threshold for the autism spectrum more frequently than females (96.9% males; 73.8% females).

The ADI-R was administered to 130 parents or caregivers. Considering the ADI-R, the significant sex differences found in the RRB subscale were disconfirmed after correcting the p value for FDR (*U* = 1269, *r* = 0.21, *p* = 0.02, adj *p* = 0.08). Only 54.3% of the sample scored above the proposed cutoff in all three main domains (communication, social interaction, restricted interests and repetitive behaviors). Again, males scored more frequently above the proposed cutoff (61.5%) than females (38.1%), even if the statistical significance of this results disappeared after correcting for FDR (*χ*^2^ = 4.42, *φ*_c_ = 0.18, *p* = 0.03, adj *p* = 0.11). The results of standardized tests for ASD are reported in Table 2.

**Table 2:** Scores obtained at the ADOS-2 and ADI-R

ADOS-2, median (range)	Total (*n* = 138)	Males (*n* = 96)	Females (*n* = 42)	*U*/*χ*^b^	*r*/φ_c_	*p* value	Adj *p* value
Communication	3 (0–8)	3 (0–8)	2 (0–6)	1582	0.18	**0.038**	0.11
Social interaction	6 (2–14)	6 (2–14)	5 (2–11)	1272.5	0.30	**0.001**	**0.008**
Communication + Social interaction	9 (2–20)	9 (2–20)	7 (2–16)	1324.5	0.27	**0.001**	**0.006**
Imagination/Creativity	1 (0–2)	1 (0–2)	0.5 (0–2)	1620	0.17	0.05	0.11
RRB	1 (0–4)	1.5 (0–4)	1 (0–4)	1400.5	0.25	**0.003**	**0.01**
ADOS-2 above the cutoffs^a^, *n* (%)	124 (89.8)	93 (96.9)	31 (73.8)	17.05	0.35	** < 0.001**	** < 0.001**

ADI-R, median (range)	Total (*n* = 130)	Males (*n* = 93)	Females (*n* = 37)	*U*/*χ*^b^	*r*/*φ*_c_	*p*-value	Adj *p*-value
Social Interaction	12 (1–30)	13 (1–30)	10 (4–27)	1407	0.14	0.10	0.18
Communication	9 (3–22)	9 (4–22)	9 (3–21)	1337	0.17	0.05	0.10
RRB	5 (1–12)	5 (1–12)	4 (1–11)	1269	0.21	**0.02**	0.08
Developmental abnormalities	1 (0–5)	2 (0–5)	1 (0–5)	1478.5	0.11	0.20	0.29
ADI-R above the cutoffs^b^, *n* (%)	75 (54.3)	59 (61.5)	16 (38.1)	4.42	0.18	**0.03**	0.11

*ADOS-2* Autism diagnostic Observation Schedule-2, *ADI-R* Autism Diagnostic Interview–Revised, *Adj p value *Benjamini–Hochberg adjusted *p* value, *C + SI *Communication + Social interaction, *RRB* restricted interests and repetitive behaviors. Significant p values and adjusted p values are marked in bold

^a^ADOS-2: A subject met the cutoff values for the autism spectrum if scores were 2 or above in the Communication domain, 4 or above in the Social Interaction domain, and 7 or above in the Communication + Social Interaction domain

^**b**^ADI-R: A subject met the cutoff values for autism if scores were 10 or above in the Social Interaction domain, 8 or above in the Communication domain (7 for non-verbal subjects), and 3 or above in the RRB domain

### Psychiatric history

As previously stated, our sample received the first formal ASD diagnosis at the median age of 23 years, with a significant difference between males and females (*U* = 1700, *r* = 0.29, *p* < 0.001, adj *p* = 0.003). Women have indeed obtained an ASD diagnosis 4 years later than men (median age of diagnosis 22 years in males and 26 years in females). 81% of males and 68% of women already had contact with mental health services. The median time elapsed between the first clinical evaluation (by a psychologist or a psychiatrist) and the ASD diagnosis was 11 years in males and 12 in females, with no statistically significant differences (*U* = 2401.5, *r* = 0.08, *p* = 0.29, adj *p* = 0.35). In particular, the first evaluation by mental health services was performed at the median age of 11.5 in males and 19 years in females. Even if we did not find a statistically significant difference (*U* = 2251, *r* = 0.12, *p* = 0.11, adj *p* = 0.18), this finding may indicate that the first evaluation in women was delayed.

33.5% of our sample had never received any psychiatric diagnosis before our evaluation. 59 people had received one psychiatric diagnosis other than ASD, 40 people two or three previous diagnoses, and 6 people four or five previous diagnoses. Notably, one woman had received six different diagnoses, and one man eight previous diagnoses (alone or in comorbidity) by other clinicians. No sex differences were found in the number of previous psychiatric diagnoses (*U* = 2513, *r* = 0.05, *p* = 0.51, adj *p* = 0.53). Information regarding the psychiatric history of our sample has been reported in Table 3.

**Table 3:** Psychiatric history of adults with autism spectrum disorder (ASD)

Variable	Total (*n* = 161)	Males (*n* = 114)	Females (*n* = 47)	*U*/*χ*^2^	*r*/*φ*_c_	*p* value	Adj *p* value
Age of diagnosis, median (range)	23 (18–55)	22 (18–55)	26 (19–51)	1700	0.29	** < 0.001**	**0.003**
Previous contact with mental health services, n (%)	125 (77.6)	93 (81.6)	32 (68.1)	3.49	0.15	0.06	0.11
Age of first evaluation, median (range)	13 (1–50)	11.5 (1–50)	19 (1–45)	2251	0.12	0.11	0.18
Gap between first evaluation and diagnosis, median (range)	11 (0–39)	11 (0–38)	12 (0–39)	2401.5	0.08	0.29	0.35
Participants with previous psychiatric diagnoses, n (%)	107 (66.5)	75 (65.8)	32 (68.1)	0.08	0.02	0.78	0.78
No. of previous diagnoses, median (range)	1 (0–8)	1 (0–8)	1 (0–6)	2513	0.05	0.51	0.53
One	59 (36.6)	43 (37.7)	16 (34)				
Two or three	40 (24.8)	26 (22.8)	14 (29.8)				
Four or five	6 (7.7)	5 (4.4)	1 (2.1)				
Six, seven or eight	2 (1.2)	1 (0.9)	1 (2.1)				

*Adj p value* Benjamini–Hochberg adjusted *p* value. Significant *p* values and adjusted *p* values are marked in bold

### Previous psychiatric diagnoses received by participants

Considering the overall sample, patients were most frequently diagnosed with ID. Of note, after administering proper standardized tests, ID was confirmed in 26 out of 35 cases, while the remaining nine patients were found having an IQ within the normal range. Other frequent diagnoses were schizophrenia and other psychotic disorders (16.1%), personality disorders (14.9%), particularly personality disorder not-otherwise specified (*n* = 16), schizoid (*n* = 4), borderline (*n* = 4), and schizotypal (*n* = 1). 13.7% of the sample had received a diagnosis of depression, and 7.5% of anxiety disorders, OCD, and conduct disorders. The 6.8% was previously identified as having ADHD. A lower number of participants were previously diagnosed with gender identity disorder (3.1%), language disorder (2.5%), bipolar disorder (2.5%), and eating disorders (1.9%). Other psychiatric disorders (e.g., substance abuse, Tourette’s syndrome, mixed developmental disorder, selective mutism) were previously identified in 4.3% of our sample.

**Fig. 1:**
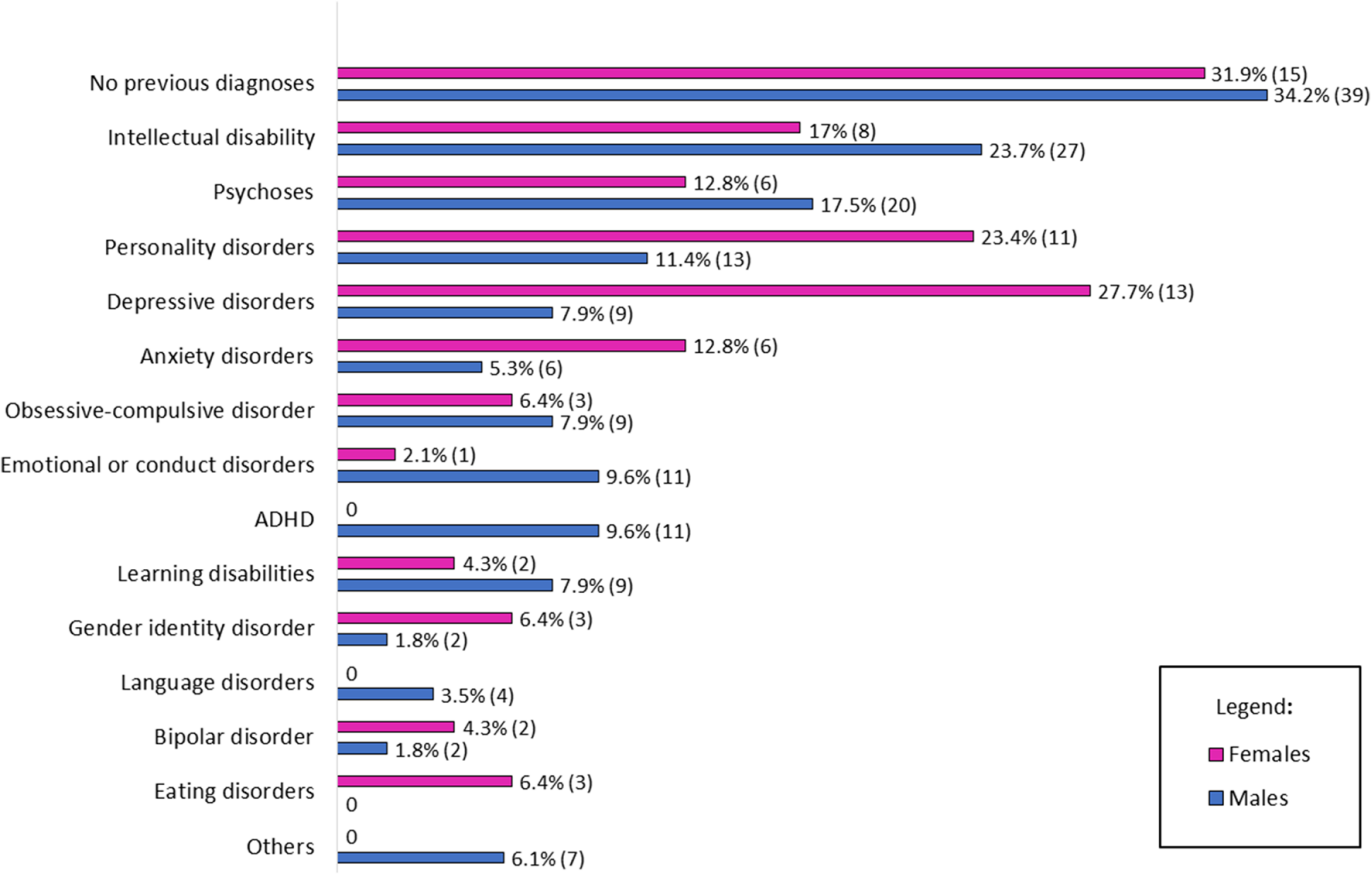
Proportion (%) and raw numbers (*n*) of past psychiatric diagnoses in adult males and females with ASD. *ADHD* attention deficit-hyperactivity disorder

Since the sample size was not sufficiently large, a statistical evaluation of sex differences in past diagnoses was not performed. However, visual inspection of Fig. 1 displays some differences between males and females. For instance, depressive and anxiety disorders, eating disorders, and personality disorders were more frequently diagnosed in women. On the other hand, males with ASD had more frequently received diagnoses of ADHD, ID, psychoses and emotional or conduct disorders. We also verified if past psychiatric diagnoses were confirmed during our assessment as comorbidities. This was true for only 52 patients (48.6% of those who had received another diagnosis).

## Discussion

### General findings

Several changes have occurred in ASD diagnostic criteria over the last decades. Originally considered a prerogative of child psychiatry, only in relatively recent years clinicians and researchers have acknowledged the lifelong persistence of the condition. In 2013, the publication of the DSM-5 has represented a substantial revolution, thanks to the introduction of the concept of “autism spectrum” and the possibility to diagnose the condition later in life [4]. Along with changes in psychiatric nosography, the awareness of ASD among the general population, parents, and mental health professionals has rapidly grown, leading to a dramatic increase in the estimated prevalence worldwide [1]. Early detection has significantly contributed to this rise in diagnoses; however, part of the growing number of cases of autism could be attributed to the identification of the so-called “lost generation”, who has started to seek a first formal diagnosis after the release of the DSM-5 [5].

The present study has been specifically designed to summarize the characteristics of people whose neurodiversity has never been identified or has been misdiagnosed with other psychiatric diseases. Our findings show that individuals who received the first diagnosis in adulthood (≥ 18 years) had generally average or above-average cognitive abilities and needed low or medium levels of support. One important issue to discuss is the long time elapsed between the first clinical evaluation and the definitive diagnosis in this specific group of people (median 11 years). This finding reflects the difficulties encountered by both child and adult psychiatrists in identifying ASD, at least in Italy. Nevertheless, our data are in line with that of Geurts and Jansen [15], who reported a median time of 12 years between the first contact with mental health services and the assessment for ASD in The Netherlands.

The proportion of psychiatric comorbidities detected during the assessment was lower compared to literature findings: for instance, anxiety disorders were experienced only by 6.2% of our sample, in contrast to 20% reported by a recent meta-analysis [14]; analogously, 9.9% of our sample had depression, in contrast to 11% found by Lai et al. [14]. Importantly, the prevalence of reported lifetime co-occurring conditions is even higher in other studies involving only participants with IQ in the normal range [26, 27].

The relatively low rate of psychiatric comorbidities found in our sample might represent one possible explanation for the late ASD diagnosis. We could hypothesize, in fact, that in the absence of the additional impairments caused by mental health disorders, individuals might have been less prone to contact our centers earlier. Another potential reason is that the limited time available for psychiatric assessment in routine clinical practice was not enough to fully understand the complex clinical pictures. Additionally, standardized tools were not systematically administered, and mainly used for differential diagnosis purposes. It is also worth mentioning that 20.5% of participants had comorbid ID, and it is well known that identifying comorbid psychopathology in people with low IQ is quite difficult, even for expert clinicians [28].

In this regard, many participants included in our sample had received a diagnosis of ID in the past, even if a comorbid diagnosis of ASD had not been performed. This is not surprising, because of the overlapping symptomatology between the two conditions: for instance, both neurodevelopmental disorders may lead to significant language impairments and may present with routine and stereotypic behaviors. Moreover, we could hypothesize that clinicians not familiar with autism (especially, those working in community mental health services) might not feel sufficiently self-confident to diagnose ASD in the presence of severe or profound ID. Interestingly, nine individuals who had received a diagnosis of ID in the past were collocated in the average or above-average range of intelligence after administering proper standardized tools. This could be due to the widespread malpractice of attributing low IQ to individuals who show excellent cognitive abilities in several non-verbal areas, despite presenting with severe socio-communication difficulties.

Adults with ASD had also received a high rate of diagnoses of psychotic disorders (16.1%) and personality disorders (PD) (14.9%), particularly “not-otherwise specified”. Like ASD, psychotic disorders can be characterized by social isolation, socially inappropriate behaviors, and low social insight. Additionally, thought disorders and the use of an atypical or nonsensical language (e.g., tangentiality, circumstantiality, neologisms) are common to both psychoses and ASD [29, 30]. PD can also have similar manifestations, such as odd behaviors (schizotypal PD), social withdrawal (schizoid PD), emotional dysregulation and self-injurious behaviors (borderline PD), low empathic traits (antisocial PD), social avoidance (avoidant PD) or sameness (obsessive–compulsive PD) [31]. Also, it is interesting to mention that past diagnoses of psychoses and PD were mainly “not-otherwise specified”. This may highlight the difficulties encountered by clinicians in framing people within specific diagnostic categories, due to heterogeneous phenotypical presentations.

In 13.7% of participants, a diagnosis of depression had been previously performed, probably for the social withdrawal [32]. Other frequent past diagnoses were anxiety disorders and OCD (7.5% each). Anxiety disorders, such as social phobia, generalized anxiety disorder (GAD), or agoraphobia, for instance, could significantly impact on the social functioning of an individual [33]. Also, OCD shares some features with ASD, such as the presence of rituals, rigid and stereotyped behaviors, or restricted interests. However, while in OCD the repetitive behaviors generally represent a means of calming obsessions and anxiety, in ASD stereotypies are usually not associated with obsessive thoughts [34]. Moreover, contrariwise to obsessions and compulsions, restricted and repetitive behaviors are not egodystonic and can play a positive function of pleasure and enjoyment, calming, or stimming, which may represent a coping strategy for people with ASD [35].

Finally, hyperactivity and behavioral problems might be interpreted as symptoms of ADHD or conduct disorders rather than expressions of the autistic condition per se, as in a small proportion of our sample. In fact, ADHD symptoms seem common in ASD; on the contrary, autistic symptoms are not common in ADHD [36]. Shared features may be, for instance, difficulties in social interaction and over-reactivity, meltdowns, or aggressive behaviors. Contrariwise, symptoms such as unusual fascination for specific interests, repetitive movements, regression of language, and special abilities are not typical of individuals with ADHD [36]. It is important to take into account that ASD and ADHD frequently co-occur [37] and this may be relevant for prognosis, since this subgroup is more likely to have quantitatively more and more severe psychiatric difficulties [38]. However, this comorbidity was not found in the sample included in this study. Our result could be partially justified by the fact that the intensity of ADHD symptoms, especially hyperactivity and impulsivity, tend to decrease during the life course [39, 40].

### Sex differences

Our findings showed that females usually obtained a more delayed diagnosis than males (26 years vs. 22 years). This is in line with the notion of the existence of a “female autism phenotype”, which has been thoroughly discussed by experts over the last few years [8, 41, 42]. The “female autism phenotype” consists in a slightly different presentation of the core and associated autistic characteristics, which may not be fully explained by the diagnostic criteria and tools, which are based on the typical male features [42]. This peculiar phenotype may partially explain the male-to-female ratio in ASD [12]. It has been widely reported that women with ASD, especially those with high cognitive abilities (such as women included in our sample), develop more effective strategies to “camouflage” difficulties in social situations [8, 43] and usually present with less pronounced symptoms, encountering the risk of going undiagnosed. Particularly, camouflaging of the autistic core deficits in females with ASD has been related to better language and social mimicry skills [44], more “active but odd” interactional behavior, less challenging behaviors or hyperactivity in the school environment, and less eccentric special interests [42].

Also, women on the autism spectrum present more frequently uncontrollable mood and interpersonal problems, as well as higher borderline and passive–aggressive traits [45, 46]. Compared to their male counterparts, they are more vulnerable to suffer from “internalizing” problems, such as anxiety, depression and eating disorders, and are less likely to present “externalizing” behaviors, such as hyperactivity, impulsivity, and conduct problems [8, 47]. This last consideration perfectly fits with our findings: indeed, women had more frequently received diagnoses of depressive, anxiety, or personality disorders that typically manifest with internalizing symptoms; conversely, males were more frequently identified as having ADHD, psychoses, or conduct disorders. However, these observations were based only on the visual inspection of Fig. 1, as we did not statistically compare the female and male data due to the small sample sizes.

Of note, while observing the general characteristics of the sample, we can notice that the ADOS-2 scores (i.e., direct observation of the patient) of the Social Interaction, Communication + Social Interaction, and RRB domains were significantly lower in the female group. We also found that 96.9% of males scored above the proposed cutoff of the ADOS-2, in contrast to only 73.8% of females, further confirming that symptoms are less pronounced in adult women. This finding is not surprising since diagnostic and screening tests, such as the ADOS-2, have been developed based on the typical male phenotype of ASD, excluding some of the features of girls with autism [41, 48]. Indeed, in one of the first studies focusing on sex-specific profiles of core symptoms in ASD diagnosed in adulthood, Lai et al. [49] found milder symptoms in females in all core areas, as assessed with the ADOS-2. The same differences were found by subsequent research [41].

Looking at the ADI-R scores (i.e., a semi-structured interview which mainly refers to childhood symptomatology), a significant difference could be detected only in the RRB domain, even if the difference disappeared after correcting for FDR. Even if a cautious interpretation is needed, our findings may support the notion that females with ASD manifest less repetitive and stereotyped behavior (RRB) than male peers, even in the presence of equal socio-communication impairments [47, 50]. The lack of associated sex-specific differences in social communication reported for early childhood, as determined by the ADI-R, may reflect the higher capability of females with ASD to develop adaptive compensation strategies beyond childhood and adolescence rather than innate behavioral differences. Indeed, both clinical observations and self-reports have suggested that females are particularly motivated and/or skilled in “camouflaging” their social difficulties [51–54].

### Limitations

To our knowledge, this is the largest study specifically focused on the characteristics and the psychiatric history of adults who received a diagnosis of ASD in adulthood. However, some limitations should be acknowledged. First, since this was a naturalistic study, our sample was probably not sufficiently large to detect all shades of this relatively new field of research; however, we have planned to extend our sample and replicate our findings. Second, differently from previous similar studies [7, 15], we focused only on adults who did obtain a diagnosis of ASD after being referred to our centers; conversely, we did not report information about individuals who asked for an ASD assessment without obtaining a final diagnosis. Since this is a crucial topic, we have planned to discuss the theme of the correctness of self-diagnosis in future studies. We believe that the main strength of our paper relies on the inclusion of people who were diagnosed with ASD after a complex clinical assessment, using standardized tools, as suggested by international guidelines. Conversely, psychiatric comorbidities (excluding ID) and adaptive abilities were not systematically assessed using standardized tools, but only performed according to the DSM-5 criteria, and we acknowledge this point as a limitation of our study. Finally, our analysis was limited to the experience of two Italian university centers. Therefore, we cannot generalize our findings to other countries, in which the awareness for the condition might be greater, and early detection of autism might be widespread. However, up to date, very few research reports have addressed the issue of undiagnosed or misdiagnosed adults with ASD presenting data from clinical practice.

### Clinical implications

Our data might be relevant for both child and adult psychiatrists, with several implications for diagnosis and treatment. On the one hand, child psychiatrists should avoid concerns in diagnosing ASD, with the risk of covering this condition under more general developmental delays. This is risky, since children with ASD may not receive specific treatments for the condition. It has in fact been demonstrated that early intervention improves both the short- and long-term outcome of ASD [5]. On the other hand, adult psychiatrists should be able to recognize the symptoms of ASD in complex psychiatric conditions, since ASD core symptoms may partially overlap those of some mental health issues (i.e., psychosis, personality disorders, ID) or be present in comorbidity. It is possible, in fact, that individuals with ASD may remain unrecognized until social demands exceed their socio-communication capacities, causing severe distress that may lead to a psychiatric consultation or hospitalization.

In case of suspected ASD, it is important to consult a specialized center for an exhaustive diagnostic assessment, which might be quite complex in adults. According to international guidelines [55] and also to our clinical experience [6], standardized tools should be administered also in adults with suspected ASD, but integrated in a complex clinical evaluation, with the consultation of caregivers, spouses, or other relatives when needed. Examining in detail the reports of past diagnoses can be useful to orientate the clinician through the diagnostic pathway.

Given the frequent misidentification of ASD with ID, also when ID is not actually present (as in nine individuals included in our sample), a standardized IQ test should be possibly administered, for several reasons. First, because it might help assessors understand whether the poor social abilities are consequences of low cognitive capacities or not, thus facilitating the differential diagnosis between ASD and ID. Second, tools examining different cognitive domains (i.e., the Weschler Scales, which compute both verbal and performance IQ) may be useful to detect some specific peculiarities of people with ASD, which might not be manifest during direct observations or interviews (e.g., deficits in fine motricity). In ASD individuals, in fact, a discrepancy between performance and verbal subtests is very common [13]. Also, people with ID usually show homogeneous impairments in cognitive profile, while ASD individuals, even those with an average intellectual functioning, tend to have scattered profiles, with areas of strengths (“islets of abilities”) and weaknesses [56]. The case of A., presented at the beginning of the paper, is quite explicative in this sense.

Apart from ID, while assessing ASD in adulthood it is important to evaluate the presence of co-occurrent conditions [57]. In fact, as reported above, the rates of psychiatric comorbidities are higher in people with ASD than the general population [14, 58]. For instance, the lifetime prevalence for adults with ASD has been estimated between 27 and 42% for anxiety disorders, and between 23 and 37% for depressive disorders [59], with peaks among individuals without ID [26, 27]. The assessment and follow-up of comorbidities have crucial implications for the outcome and the follow-up. For instance, the presence of psychiatric comorbidities may increase the risk of suicidal ideation and behaviors [60]. A recent review has reported that prevalence rates for suicidal ideation were between 11 and 66% and suicidal attempts were between 1 and 35% in ASD [61]. Also, Hirvikoski et al. [57] reported that 0.31% of premature deaths in ASD were due to suicide. Moreover, according to the study, suicide was 7.55 times higher in people with ASD than controls, and even 9 times higher in ASD people without associated ID, who often present with co-existing psychiatric disorders [57].

Getting to a diagnosis of ASD and a timely identification of comorbidities have also relevant implications for the choice of treatment. As suggested by guidelines, psychosocial and behavioral therapies should be preferred in people with ASD [55, 62]. Conversely, obsessive-like symptoms (i.e., repetitive behaviors or restricted interests), paranoia, or social withdrawal might not be responsive to common psychiatric psychopharmacological treatments, sometimes causing important side effects [63]. Moreover, as reported above, repetitive behaviors and restricted interests are typically egosyntonic and may represent fundamental coping strategies for people with ASD, having a function of calming or stimming to deal with under- or overstimulation [35]. Therefore, suppressing these behaviors might be deleterious for this group of people. On the contrary, other co-occurrent conditions (e.g., moderate to severe depression and anxiety, OCD, and psychoses above all) might be worthy of pharmacological treatment. Importantly, a diagnosis of ASD might also facilitate the achievement of specific support, such as disability benefits, or academic and job inclusion.

## Conclusion

Our data revealed that ASD is still poorly recognized by both child and adult psychiatrists, and sometimes confused with other disorders with similar phenotypical presentations. This is probably due to the lack of awareness of the condition among clinicians, but also to the complex and different phenotypes of the spectrum, which might lead to a late diagnosis. Nevertheless, given the overlapping symptoms with other disorders, and the high rates of psychiatric co-occurrent conditions in people with ASD, it is important to consider the possibility of an ASD diagnosis in adults who are referred to mental health services. However, a careful assessment made by psychiatrists who are expert in the field needs to be conducted. The identification of ASD also in the adult population is crucial for adequate planning of treatment and global case management.
